# Phylogenetic Diversity of Archaea and the Archaeal Ammonia Monooxygenase Gene in Uranium Mining-Impacted Locations in Bulgaria

**DOI:** 10.1155/2014/196140

**Published:** 2014-03-11

**Authors:** Galina Radeva, Anelia Kenarova, Velina Bachvarova, Katrin Flemming, Ivan Popov, Dimitar Vassilev, Sonja Selenska-Pobell

**Affiliations:** ^1^Helmholtz-Centre Dresden-Rossendorf, Institute of Resource Ecology, Bautzner Landstraße 400, 01328 Dresden, Germany; ^2^Institute of Molecular Biology, Bulgarian Academy of Sciences, Academic G. Bonchev Street, Building 21, 1113 Sofia, Bulgaria; ^3^Faculty of Biology, Sofia University, 8 Dragan Tsankov Boulevard, 1164 Sofia, Bulgaria; ^4^Molecular Medicine Centre, Medical University of Sofia, 1 Georgy Sofiiski Street, 1431 Sofia, Bulgaria; ^5^Agrobioinstitute, 8 Dragan Tsankov Boulevard, 1164 Sofia, Bulgaria

## Abstract

Uranium mining and milling activities adversely affect the microbial populations of impacted sites. The negative effects of uranium on soil bacteria and fungi are well studied, but little is known about the effects of radionuclides and heavy metals on archaea. The composition and diversity of archaeal communities inhabiting the waste pile of the Sliven uranium mine and the soil of the Buhovo uranium mine were investigated using 16S rRNA gene retrieval. A total of 355 archaeal clones were selected, and their 16S rDNA inserts were analysed by restriction fragment length polymorphism (RFLP) discriminating 14 different RFLP types. All evaluated archaeal 16S rRNA gene sequences belong to the 1.1b/*Nitrososphaera* cluster of Crenarchaeota. The composition of the archaeal community is distinct for each site of interest and dependent on environmental characteristics, including pollution levels. Since the members of 1.1b/*Nitrososphaera* cluster have been implicated in the nitrogen cycle, the archaeal communities from these sites were probed for the presence of the ammonia monooxygenase gene (*amo*A). Our data indicate that *amo*A gene sequences are distributed in a similar manner as in Crenarchaeota, suggesting that archaeal nitrification processes in uranium mining-impacted locations are under the control of the same key factors controlling archaeal diversity.

## 1. Introduction

Metagenomic studies have revealed that Archaea are widely distributed and likely play an important role in a variety of environmental processes, such as chemoautotrophic nitrification [1], carbon metabolism [2], and amino acid uptake [3, 4]. The most abundant organisms among the archaeal phyla are Crenarchaeota and Euryarchaeota [2, 5]. Crenarchaeota represent more than 75% of the archaeal populations in natural environments [6]. Certain crenarchaeotic groups are thought to be confined to specific environments; for example, group 1.1a consists mainly of aquatic organisms, while the members of group 1.1b are typical soil crenarchaeotes [7].

Worldwide mining and milling activities have introduced high levels of radionuclides and heavy metals (HMs) into soil and aquatic environments. The adverse effects of pollutants on Archaea are not well studied [8, 9]. Moreover, only a few studies have investigated archaeal diversity in HM- [10, 11] and uranium- (U-) contaminated environments [5, 12–14]. Radeva and Selenska-Pobell [13] reported crenarchaeotic 16S rRNA gene sequences in U-contaminated soils of Saxony, Germany, belonging only to the 1.1b group of the phyla, while Reitz et al. [14] identified 1.1a, 1.3b, and SAGMCG.1 crenarchaeotic gene sequences from deeper U-polluted soil horizons. Porat et al. [5] investigated the diversity of archaeal communities from mercury- and U-contaminated freshwater stream sediments by pyrosequencing analysis. They found a higher abundance and diversity of Archaea in mercury- than in U-contaminated sites, where the archaeal sequences were of both the Crenarchaeota and Euryarchaeota phyla.

To date, little is known concerning the interactions between archaea and U or HMs. Kashefi et al. [15] published that the hyperthermophilic crenarchaeote* Pyrobaculum islandicum* is able to reduce U(VI) to U(IV) under anaerobic conditions at 100°C. Francis et al. [16] demonstrated that the halophilic euryarchaeote* Halobacterium halobium* accumulates high amounts of U(VI) as extracellular uranyl phosphate deposits; however, these two organisms are not found in U-contaminated substrata. Later, Reitz et al. [9, 17] revealed the capacity of the acidothermophilic* Sulfolobus acidocaldarius*, which is an indigenous archaeon for U-contaminated soils and mine tailings, to accumulated intracellular U(VI).

The discovery that some mesophilic archaea from Crenarchaeota, which were later categorized into the new Thaumarchaeota phylum [18], have the potential to oxidize ammonia suggests an important role of archaea in the nitrogen (N) cycle [19, 20]. The crenarchaeotic ammonia monooxygenase gene (*amo*A) is found in many natural environments, such as soil [2, 21], marine, and freshwater ecosystems [22–25], several geothermal environments and hot springs [26–28], Artic lakes [29], drinking water production plants [30], and wastewater treatment plants [31]. This widespread distribution indicates the ubiquity and significance of archaeal ammonia oxidizers in the global N cycle [21, 32–34]. However, there are few studies assessing the abundance of archaeal* amo*A and its diversity in U-impacted environments.

Intensive U mining and milling in Bulgaria were performed between 1946 and 1990 and have caused significant soil and water pollution. U production was stopped by a government decree in 1992, and mines and tailings were technically liquidated and gradually remediated. Nevertheless, their surroundings are still highly contaminated, and further contamination from the compromised remediation of mines and tailings has been recorded.

The aim of this study was to investigate the diversity of archaeal communities inhabiting environments impacted by U mining and milling activities and in particular to reveal the diversity of the archaeal* amo*A gene. Since U and HM contamination represent an old environmental burden, we expected that the composition and diversity of archaeal and* amo*A communities were stabilized under the selective power of both contamination level and environmental characteristics.

## 2. Materials and Methods

### 2.1. Sites and Sampling

**Figure 1:**
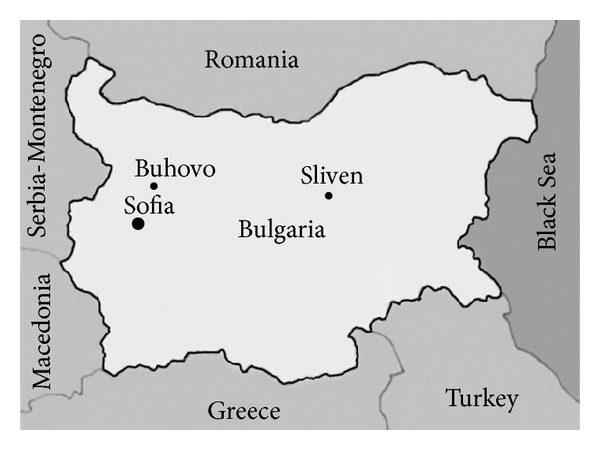
Map of Bulgaria and the location of the studied sites Buhovo (BuhC and BuhD) and Sliven (Sliv).

Two locations in Bulgaria were studied: the abandoned mining and milling complex “Buhovo” and the “Sliven” mine, both of which have been classified as areas of high radiological risk by the Bulgarian Agency for Radiobiology and Radioprotection. The mining complex “Buhovo” (42°45′51.20′′N; 23°34′36.86′′E) is located 30 km northeast of Sofia on a 2,280 ha territory, while the “Sliven” mine (42°41′47.68′′N; 26°22′22.47′′E) is located in South Eastern Bulgaria and occupies an area of 491 ha (Figure 1). Mining operations at the two locations were conducted in a conventional underground manner from 1962 to 1981. They were officially closed in 1992 and remediated until 2001.

Samples from Buhovo were collected in May 2003 at depths of 20 cm (BuhC) and 40 cm (BuhD). Samples labelled “Sliv” were collected in June 2004 from the “Sliven” mine waste pile at a depth of 40 cm. Five samples from BuhC, BuhD, and Sliv were collected under sterile conditions, transported at 4°C, and stored at −20°C until use.

### 2.2. Environmental Variables

The organic matter content of the sample was determined by Turyn's method based on its oxidation by potassium dichromate [35]. The pH was measured using a portable potentiometer (HANA pH meter) after the soil samples had been suspended in distilled water (soil : liquid, 1 : 2.5). The concentrations of sulfates and nitrates were determined using a spectrophotometer in 0.1 M CaCl_2_ soil extract following methods described by Bertolacini and Barney II [36] and Keeney and Nelson [37], respectively. The concentration of HMs was measured using an ELAN 5000 Inductively Coupled Plasma Mass Spectrometer (Perkin Elmer, Shelton, CT, USA) in a 1 M HCl solution (1 : 20; soil : 1 M HCl). The results were calculated for oven-dried soil.

### 2.3. DNA Extraction

Total DNA (>25 kb) was extracted from the samples (3 g) after direct lysis using the method described by Selenska-Pobell et al. [38], and the DNA subsamples (five DNA subsamples for sampling site) were collected in a representative average sample for further analysis.

### 2.4. PCR Amplification

Archaeal 16S rRNA genes from the genomic DNA were amplified via seminested PCR using specific archaeal 16S_21–40F_ (5′-TTCCGGTTGATCCYGCCGGA-3′) and universal 16S_1492–1513R_ (5′-ACGGYTACCTTGTTACGACTT-3′) primers. Each PCR reaction mixture (20 *μ*L) contained 200 *μ*M deoxynucleotide triphosphates, 1.25 mM MgCl_2_, 1.25 mM MgCl_2_, 10 pmol DNA primers, 1–5 ng template DNA, and 1 U AmpliTaq Gold polymerase with the corresponding 10x buffer (Perkin Elmer, Foster City, CA, USA). The amplifications were performed with a “touch down” PCR in a thermal cycler (Biometra, Göttingen, Germany). After an initial denaturation at 94°C for 7 min, the annealing temperature was decreased from 59 to 55°C over five cycles, followed by 25 cycles each with a profile of denaturation at 94°C (60 sec), 55°C (40 sec), and 72°C (90 sec). The amplification was completed by an extension of 20 min at 72°C. The diluted products of the first reaction were used as templates for the second round of PCR, where two archaeal specific primers 16S_21–40F_ and 16S_940–958R_ (5′-YCCGGCGTTGAMTCCAATT-3′) were applied [39]. The initial denaturation at 95°C for 7 min was followed by 25 cycles each consisting of denaturation at 94°C (60 sec), annealing at 60°C (60 sec), and polymerization at 72°C (60 sec). The amplification was completed by an extension of 10 min at 72°C. This seminested PCR format was applied to obtain a sufficient amount of PCR products for the cloning procedure.

Archaeal* amo*A fragments (~635 bp) were amplified using the PCR primers Arch-*amo*AF (5′-STAATGGTCTGGCTTAGACG-3′) and Arch-*amo*AR (5′-GCGGCCATCCATCTGTATGT-3′) [40]. PCR cycling was conducted according to Francis et al. [40], with an initial denaturation at 95°C for 5 min followed by 35 cycles of the following: denaturation at 94°C (45 sec), annealing at 53°C (1 min), and extension at 72°C (1 min). Amplification was completed by an extension of 15 min at 72°C.

### 2.5. 16S rRNA Gene Clone Libraries

One archaeal and one* amo*A gene clone libraries for BuhC, BuhD, and Sliv were constructed using the pooled products from the PCR reactions. The 16S rDNA amplicons from five replicates were combined and cloned directly into* Escherichia coli* using a TOPO TA Cloning Kit (Invitrogen, Carlsbad, CA, USA) following the manufacturer's instructions to generate clone libraries. The archaeal 16S rRNA gene inserts and* amo*A gene inserts were subsequently amplified by PCR with plasmid-specific primers for the vectors M13 and M13 rev and then digested (2 h, 37°C) with the* Msp*I and* Hae*III restriction enzymes following the manufacturer's instructions (Thermo Fisher Scientific, USA). Restriction fragment length polymorphism (RFLP) patterns were visualized using 3.5% Small DNA Low Melt agarose gels (Biozym, Hessisch, Oldenburg, Germany), and these data were then used to group clones into phylotypes. The representatives of the RFLP types were purified using an Edge BioSystems Quick-Step 2 PCR Purification Kit (MoBiTec, Gottingen, Germany) and then sequenced using the BigDye Termination v.3.1 Kit (Applied Biosystems) and ABI PRISM 310 DNA sequencer (Applied Biosystems, Foster City, CA, USA). The sequencing of archaeal 16S rRNA gene fragments was performed using the primers 16S_21–40 F_ and 16S_940–958R_, while* amo*A gene fragments were sequenced using the vector primer SP6.

### 2.6. Phylogenetic Analysis

The sequences obtained were analysed and compared with those in the GenBank database using the BLAST server at the National Centre for Biotechnology Information (NCBI) (http://www.ncbi.nlm.nih.gov). The presence of chimeric sequences in the clone libraries was determined using the programs CHIMERA CHECK, available on the Ribosomal Database Project II (release 11.0) and Bellerophon [41]. The sequences were aligned with those corresponding to the closest phylogenetic relatives using the Clustal W program [42]. Phylogenetic trees were constructed according to the neighbour-joining method using the Bioedit software package.

### 2.7. Data Analysis

The results were statistically analysed by NCSS97 (NCSS, Kaysville, Utah), and the average values were presented. The sampling efficiency and diversity within the archaeal clone libraries were estimated using the MOTHUR software program based on the furthest-neighbour algorithm, and the sequences were grouped into operational taxonomic units (OTUs) [43] at sequence similarity levels (SSLs) of BuhC ≥ 97% (0.03 distance), BuhD ≥ 94% (0.06 distance), and Sliv ≥ 91% (0.09 distance). For each sample, the archaeal OTU richness (rarefaction curves, Chao 1, ACE) [44] and diversity (Shannon-Weiner index) [45] estimates were calculated. Statistical analysis of* amo*A OTUs was not carried out because of the low number of unique gene sequences identified in the BuhC, BuhD, and Sliv clone libraries. The level of pollution was expressed using a toxicity index (TI) as follows:

(1) $$\begin{array}{c} \text{TI}=\frac{\sum C_{i}}{{\text{ED}50}_{i}}, \end{array}$$

where *C*_*i*_ is the concentration of metal *i* in substratum (mg kg^−1^) and ED50 is the total concentration of metal causing 50% reduction in microbial dehydrogenase activity (original ED50s were taken from Welp [46]).

### 2.8. Nucleotide Sequence Accession Numbers

The sequences reported in this study were deposited in GenBank under the following accession numbers: FM897343 to FM897356 for partial archaeal 16S rRNA gene sequences and FM886822 to FM886831 for crenarchaeotic* amo*A gene sequences.

## 3. Results

### 3.1. Environmental Variables

Buhovo and Sliven samples differed in their geochemistry and the levels of U and HM contamination. BuhC and BuhD were sampled (Chromic cambisols) from different soil depths, while Sliv was a sandy gravel material collected from a mine waste pile. The texture of BuhC (20 cm at soil depth) was classified as sandy clay (35% silt and 54% clay), whereas BuhD (40 cm at soil depth) was classified as clay (38% silt and 60% clay). The bulk density of Buh soil varied in depth from 1.5-1.6 g cm^−3^ (20 cm) to 1.7-1.8 g cm^−3^ (40 cm). Soil porosity was 36–40% (20 cm) and 25–30% (40 cm) (personal communication). There is no data concerning the texture and geochemistry of Sliv substratum, except the organic matter content (0.3%) and pH (7.5). The organic matter content of the Buh samples was 2.8% for BuhC and 1.6% for BuhD. The total amount of nitrogen decreased from 1.19 g kg^−1^ (20 cm) to 1.03 g kg^−1^ (40 cm), while the total amount of phosphorus was not significantly different between the two soil layers—0.53 g kg^−1^ (20 cm) and 0.51 g kg^−1^ (40 cm). The pH_H_2_O_ of BuhC and BuhD was slightly acidic (pH 6.9 and 6.6, resp.).

The main pollutants were Cu and Zn (BuhC, BuhD, and Sliv), U (BuhC and Sliv), Cr (BuhC and BuhD), As (BuhC and Sliv), Pb (Sliv), and sulfates (BuhD) (Table 1). All sites were highly contaminated as shown by their individual TI_*i*_ (*i*—heavy metal with TI > 1.0) and TI_sum_, which decreased as follows: Sliv (119.38) > BuhC (15.38) > BuhD (9.91). Moreover, the level of toxicity might actually be stronger if the values took into account Mn (BuhC and BuhD) and U (BuhC and Sliv), since their concentrations were also high. However, the TI_sum_ did not include these due to a lack of ED50 data.

### 3.2. Phylogenetic Diversity of Archaeal and* amo*A Gene Sequences

**Figure 2:**
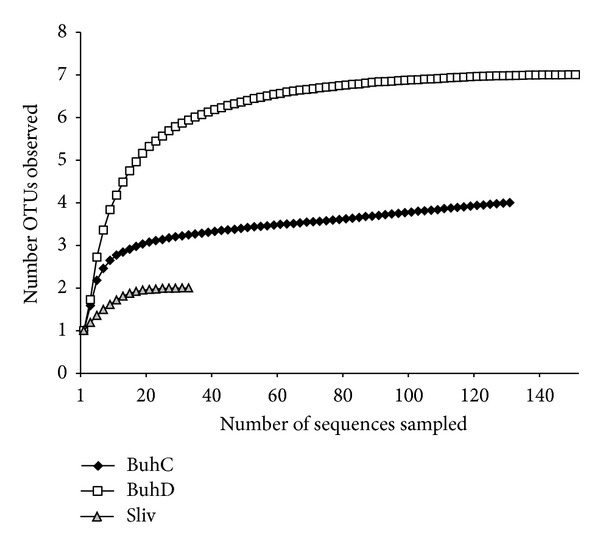
Rarefaction curves indicating archaeal 16S rRNA richness within BuhC (SSL 97%), BuhD (SSL 94%), and Sliv (SSL 91%) clone libraries.

A total of 355 archaeal clones (156 from BuhC, 128 from BuhD, and 71 from Sliv) and 229* amo*A gene clones (107 from BuhC, 99 from BuhD, and 23 from Sliv) were selected, and their 16S rDNA inserts were analysed by RFLP. The clones sequenced were grouped into 19 (archaeal) and 15 (*amo*A) OTUs, and out of these 14 OTUs and 10 OTUs were unique, respectively. The rarefaction curves of the archaeal BuhC (3.99 ± 0.24 OTUs), BuhD (6.99 ± 0.07 OTUs), and Sliv (1.99 ± 0.06 OTUs) clone libraries were saturated, indicating that they completely covered the natural archaeal diversity of the samples and that the observed OTUs were a good representation of the archaeal community richness (Figure 2). The estimates of archaeal richness (Chao 1, ACE) and diversity (Shannon-Weiner index) predicted the highest values of indices in BuhD, followed by the BuhC and Sliv clone libraries (Table 2).

### 3.3. Archaeal Community Composition

**Figure 3:**
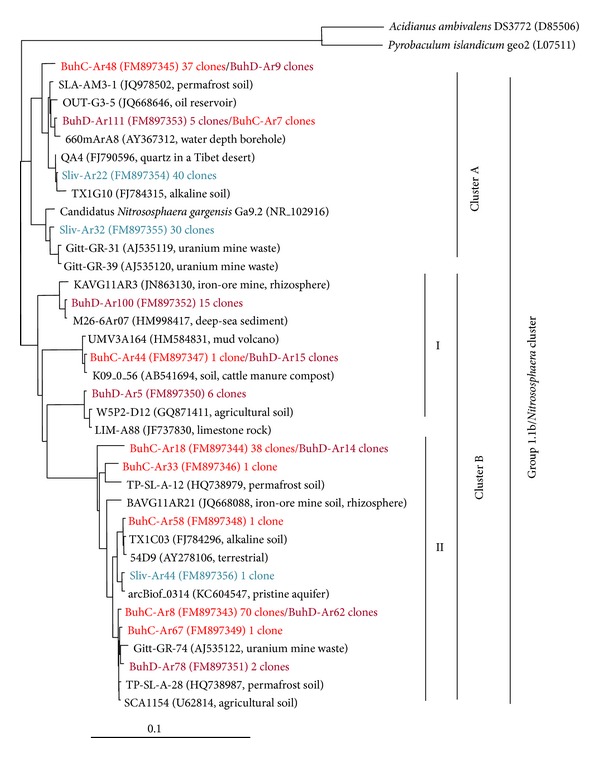
Phylogenetic analysis of archaeal 16S rRNA gene sequences retrieved from uranium mining sites BuhC, BuhD, and Sliv. The tree was constructed using the neighbour-joining method. The 16S rRNA sequences of* Acidianus ambivalens* DS3772 and* Pyrobaculum islandicum* geo2 were used as an outgroup. The scale bar represents 0.1 changes per nucleotide position.

The 16S rRNA gene sequences identified in BuhC, BuhD, and Sliv belonged to the 1.1b/*Nitrososphaera* cluster of Crenarchaeota (Figure 3). Representatives of other crenarchaeotic clades or other archaeal phyla were not detected in this study.

The crenarchaeotic sequences were grouped into clusters (A and B; Figure 3). Cluster A involved 16S rRNA gene sequences retrieved mainly from the highly polluted environments of Sliv and BuhC. Cluster B consisted of OTUs from the BuhC and BuhD (226 of 227 clones) libraries. The latter cluster was separated into subcluster IB, generated by the sequences of the BuhD clone library (36 of 37 clones), and subcluster IIB, which mainly consisted of clones belonging to the BuhC and BuhD libraries (190 of 196 clones).

There were common (BuhC-Ar8, BuhC-Ar18, BuhC-Ar48, and BuhD-Ar111) 16S rRNA gene archaeal sequences in the clone libraries of BuhC and BuhD. We did not retrieve any gene sequences common to the Sliv and Buh substrata.

All retrieved 16S rRNA gene sequences matched to sequences of uncultured archaea, except Sliv-Ar32, which was affiliated with the cultured archaeon* Candidatus Nitrososphaera gargensis* (NR_102916).

### 3.4. Composition of the* amo*A Community

**Figure 4:**
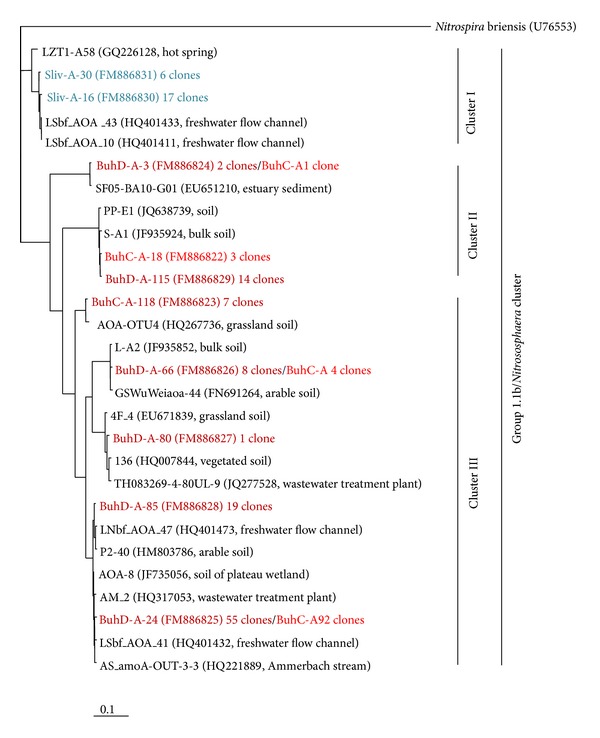
Phylogenetic analysis of archaeal* amo*A gene sequences retrieved from uranium mining sites BuhC, BuhD, and Sliv. The tree was constructed using the neighbour-joining method. The* amo*A sequence of* Nitrospira briensis* was used as an outgroup. The scale bar represents 0.1 changes per nucleotide position.

Phylogenetic analysis of 10 archaeal* amo*A OTUs revealed a high sequence identity (98–100%) with ammonia-oxidizing crenarchaeotes. Cluster I from the phylogenetic tree of the* amo*A gene sequences was formed by two OTUs from Sliv, whereas clusters II and III were only composed of OTUs from the Buhovo soil environments (Figure 4). In total, all* amo*A OTUs were presented in a relatively small number of clones (1–15 clones), except BuhD-A-24 and its analogue OTU from BuhC, which consisted of 55 and 92 clones, respectively.

All retrieved archaeal* amo*A sequences were matched with uncultured crenarchaeotes.

Protein sequences derived from the same samples were also analysed, and the data validated our DNA results (data not published). The protein sequences exhibited 96–100% similarity to the closest matched GenBank sequences retrieved from terrestrial, estuarine, and hot spring environments.

## 4. Discussion

The BuhC, BuhD, and Sliv archaeal communities appear to be composed solely of members of the soil-freshwater-subsurface group (1.1b) of Crenarchaeota, which was recently assigned by Bartossek et al. [49] as* Nitrososphaera* cluster. The presence of Crenarchaeota in these sites was not surprising, since these organisms are widespread [4, 7, 50], even in environments highly polluted with U and HMs [5, 7, 13, 51]. Probably, the selection and propagation of only 1.1b Crenarchaeota in Buhovo and Sliven are passed under the power of U and HM pollution. Supporting this notion, Geissler et al. [52], Reitz et al. [14], and Radeva et al. [53] reported a strong reduction in archaeal diversity and a shift from Crenarchaeota 1.1a to 1.1b in soil samples supplemented with uranyl nitrate. The adverse effects of U were also confirmed by Porat et al. [5], who found low archaeal diversity in U-/nitrate-contaminated sediments of the Oak Ridge stream (TN, USA).

The importance of the substratum and the level of pollution in the pattern of crenarchaeotic distribution is evident from the archaeal phylogenetic tree (Figure 3), where OTUs are grouped in one large cluster (B) based on 16S rRNA gene sequences from Buhovo soil (9 of 10 OTUs/226 of 227 clones) and another smaller cluster (A) formed of OTUs from the most polluted environments, Sliv and BuhC (4 of 6 OTUs/114 of 128 clones). There are no common 16S rRNA gene sequences from the two substrata (Buh soil and Sliv sandy gravel matter) studied.

The distinct physical and geochemical niches of the sites harbour characteristic crenarchaeotic populations (Figure 3): (i) typical soil species tolerant towards environmental extremes, including resistance to U and HMs (members of subcluster IIB); (ii) depth specific species, probably, sensitive to U and HMs (members of subcluster IB); and (iii) resistant to U and HM soil and rocky inhabitants (cluster A). All OTUs correspond to terrestrial environmental matches, except Sliv-Ar44, BuhD-Ar100, and BuhD-Ar111, which exhibit high similarity (99-100%) with gene sequences derived from aquatic environments: groundwater (KC604547), deep-sea sediments (HM998417), and seawater at depths of 660 m (AY367312), respectively. In general, the above-mentioned water-related OTUs are only represented by a small number of clones (1–15).

The Buh soil environments comprise more complex and more diverse archaeal communities: 84% of OTUs and 80% of archaeal clones are from Buh, which validates data from Ochsenreiter et al. [7] indicating that the 1.1b crenarchaeotic clade is a typical “soil lineage.”

Archaeal diversity in Buh soil is relatively low, varying from 0.97 (BuhC) to 1.51 (BuhD), and is depth dependent. Archaeal communities of the two soil depths include both common (BuhC-Ar8, BuhC-Ar18, BuhC-Ar44, BuhC-Ar48, and BuhD-Ar111) and depth-specific 16S rRNA gene sequences, the latter of which are represented by a small number of clones (1–15 clones). The dominant OTU BuhC-Ar8 is equally distributed in soil depth, comprising 45% and 48% of clones retrieved from BuhC and BuhD, respectively. Moreover, it is closely affiliated (99% SSL) with the uncultured crenarchaeote Gitt-GR-74 (AJ535122), which is found in uranium mill tailing in Saxony, Germany [13].

A trend for depth dependency in archaeal distribution was also observed in other studies, which indicate that Crenarchaeota are more abundant in deeper soil layers [54–57] and that archaeal : bacterial ratios increase with soil depth [2]. In the aforementioned studies, increasing abundance of crenarchaeotes correlated with decreasing nutrient (organic carbon and inorganic nitrogen) and oxygen concentrations in deeper soil layers. In agreement with the above-mentioned statements, we can speculate for BuhD that the diversity of Crenarchaeota is favoured by the nutritional and oxygen status of this soil depth and its low levels of U and HM pollution. The relative opposite conditions in BuhC soil layer comparing to BuhD (higher organic matter content, higher aeration in the upper soil layer, and higher levels of U and HMs) limit its archaeal diversity mainly to three dominant OTUs (BuhC-Ar8, BuhC-Ar18, and BuhC-Ar48) that harboured 93% of clones in the BuhC clone library.

The sandy gravel substratum of Sliv and its high level of pollution make this environment very unfavourable for archaeal proliferation. The inhabitants of Sliv are presented by two main OTUs (Sliv-Ar32 and Sliv-Ar22) that comprise 99% of clones. All archaeal 16S rRNA gene sequences retrieved from Sliv correspond with uncultured crenarchaeotic matches, except Sliv-Ar32, which exhibits a 99% similarity with* Candidatus Nitrososphaera gargensis* Ga9.2. According to Spang et al. [58],* Ca. N. gargensis* is well adapted to HM-contaminated environments and encodes a number of HM resistance genes that convey the genetic capacity to respond to environmental changes. The close similarity of Sliv-Ar32 to Gitt-GR sequences (99% SSL) recovered from U mill tailings in Germany also confirms the high tolerance of Sliv-Ar32 towards U and HM pollution. The other, more abundant OTU is Sliv-Ar22 (40 clones), and its dominance in Sliv clone library can be explained by both tolerance towards high levels of pollution and ability of Sliv-Ar22 archaeon to colonize rocky substrata. This sequence exhibits high similarity to the uncultured crenarchaeote QA4 (99% SSL), which was recovered from quartz rocks located in the high-altitude tundra of Central Tibet [59].

The phylogenetic analysis of archaeal* amo*A gene sequences retrieved from BuhC, BuhD, and Sliv reveals that the Crenarchaeota inhabiting these locations harbour ammonia oxidizers (Figure 4). The pattern of* amo*A gene sequence distribution is similar to that of Crenarchaeota with the smallest number of OTUs in the most unfavourable environment of Sliv (2 OTUs/23 clones), followed by the highly polluted BuhC (5 OTUs/107 clones) and the relatively low polluted BuhD (6 OTUs/99 clones). The high number of* amo*A OTUs in BuhD is related to the highest archaeal diversity in this depth and is due to the favourable conditions (low organic matter, nitrogen and oxygen content, and high clayey soil texture) which stimulate not only the archaeal diversity but also the diversity of ammonia-oxidizing archaea. To date, studies [33, 60–63] that have investigated the environmental factors that shape* amo*A gene diversity in oceans, sediments, and soils have identified these factors as key environmental parameters for the proliferation of ammonia-oxidizing archaea.

Forty-six percent of the archaeal* amo*A OTUs, which comprise 73% of clones retrieved in this study, affiliate with archaeal* amo*A gene sequences obtained from freshwater ecosystems [64, 65] and wastewater treatment plants [66]. These belong to the “soil and other environments” cluster, as proposed by Prosser and Nicol [67]. The other* amo*A OTUs (all from BuhD and BuhC) exhibit gene sequences closely related to those retrieved from soil environments like bulk [60] and arable (FN691264, HM803786) soils, grassland (HQ267736, EU671839), and semiarid soil (JQ638739) that belong also to the “soil and other environments” cluster [67].

BuhC and BuhD are very different environments with regard to soil texture, nutrients, oxygen (low soil porosity), and pollution status. Nevertheless, the two environments are inhabited by ammonia-oxidizing archaea as determined by the presence of the* amo*A gene sequence; BuhD-A-24 comprised 23% (BuhD) and 41% (BuhC) of all retrieved* amo*A clones. It is likely that the exclusive domination of BuhD-A-24 in Buhovo soil depths is a result of the adverse effects of pollution that reduce archaeal* amo*A diversity and the selection of only a few resistant gene sequences. We did not detect novel archaeal* amo*A clusters that would indicate the existence of special U- and HM-resistant ammonia-oxidizing archaea in the sites studied. This reveals the widespread distribution of ammonia-oxidizing archaea and the capacity of some species to tolerate high levels of U and HMs.

## 5. Conclusions

Phylogenetic analysis revealed that all archaeal 16S rRNA gene sequences assessed in this study belong to the 1.1b/*Nitrososphaera* cluster of Crenarchaeota. The diversity of crenarchaeotic communities that inhabit the three sites of interest was very low, especially in the high U- and HM-polluted, sandy-stone environment of the Sliv mine. The archaeal communities of Buh and Sliv mines were distinct to each site and did not harbour common gene sequences. We did not detect novel crenarchaeotic and* amo*A gene clusters, indicating that the polluted environments of Buh and Sliv are inhabited by typical archaeal soil lineages. It is likely that these archaeal soil lineages were selected by the multifactorial nature of the local environment, resulting in the development of tolerance of indigenous archaea to high U and HM pollution. The archaeal* amo*A gene sequences detected in BuhC, BuhD, and Sliv supposed that ammonia-oxidizing archaea participate in nitrogen cycling in environments highly polluted with U and HMs. This study will be helpful in understanding the archaeal and ammonia-oxidizing archaeal diversities in soils polluted with U and HMs.

## Figures and Tables

**Table 1:** Physicochemical characteristics of samples from three sites in Bulgaria polluted by uranium mining activities, expressed as means ± standard deviation (*n* = 15).

Parameter	*D*	BC	BuhC	BuhD	Sliv
pH	—	—	6.9 ± 0.3	6.6 ± 0.2	7.5 ± 0.3
OM	%	—	2.8 ± 1.3	1.6 ± 1.0	0.3 ± 0.1
NO_3_-N	mg/kg	—	21.6 ± 12.9	9.4 ± 6.6	19.9 ± 11.0
SO_4_	mg/kg	—	786 ± 95.0	1300 ± 142.0	151 ± 14.0
As	mg/kg	3.84	274 ± 13.0^1^	72.4 ± 2.8^1^	412 ± 22.0^1^
Cd	mg/kg	0.15	2.4 ± 1.3^1^	1.1 ± 1.2	2.7 ± 1.8^1^
Co	mg/kg	ND	29.5 ± 1.2	27.2 ± 1.2	22.4 ± 1.4
Cr	mg/kg	51.00	89.6 ± 2.6	95.2 ± 7.4	8.6 ± 1.9
Cu	mg/kg	47.34	236 ± 11.4^1^	101 ± 21.0	3410 ± 87.0^1^
Ni	mg/kg	36.41	75.2 ± 13.4	98.4 ± 8.9^1^	37.0 ± 11.0
Pb	mg/kg	19.19	674 ± 39.4^1^	126 ± 16.3	5160 ± 49.9^1^
Zn	mg/kg	54.98	448 ± 52.0^1^	464 ± 23.1^1^	1270 ± 98.4^1^
U	mg/kg	0.3–11*	200 ± 21.2	78.4 ± 8.7	374 ± 11.2
TI_As_	—	—	1.63 ± 0.08	0.43 ± 0.02	2.45 ± 0.13
TI_Cd_	—	—	0.03 ± 0.01	0.01 ± 0.00	0.03 ± 0.02
TI_Co_	—	—	0.05 ± 0.00	0.05 ± 0.00	0.04 ± 0.00
TI_Cr_	—	—	1.26 ± 0.03	1.34 ± 0.1	0.12 ± 0.02
TI_Cu_	—	—	6.74 ± 0.32	2.88 ± 0.60	97.43 ± 2.50
TI_Ni_	—	—	0.75 ± 0.13	0.98 ± 0.09	0.37 ± 0.11
TI_Pb_	—	—	1.03 ± 0.06	0.19 ± 0.02	7.90 ± 0.08
TI_Zn_	—	—	3.89 ± 0.45	4.03 ± 0.00	11.04 ± 0.86
TI_sum_	—	—	15.38	9.91	119.38

^1^Value above the maximum allowable concentration referring to Bulgarian legislation [47]. *Values according to UNSCEAR [48]. ND: no data; *n*: number of samples; D: dimension; BC: background concentrations referring to Bulgarian legislation [47]; TI_sum_: sum of toxicity indices of heavy metals (except U) and metalloid As.

**Table 2:** Predicted richness (Chao 1 and ACE) and diversity (Shannon-Weiner index) of BuhC, BuhD, and Sliv 16S rDNA archaeal clone libraries, expressed as means ± standard deviation.

Clone library	Number of clones	Number of OTUs	Number of singletons/doubletons	Chao 1	ACE	Shannon-Weiner index
BuhC^a^	156	7	4	4 ± 0.25	N/A	0.97 ± 0.10
BuhD^b^	128	8	1	7 ± 0.00	7 ± 0.00	1.51 ± 0.13
Sliv^c^	71	3	1	2 ± 0.00	2 ± 0.00	0.32 ± 0.24

OTUs were defined at ^a^3%, ^b^6%, and ^c^9% differences in 16S rRNA gene sequences.
